# Expanding the clinical spectrum associated with defects in *CNTNAP2 *and *NRXN1*

**DOI:** 10.1186/1471-2350-12-106

**Published:** 2011-08-09

**Authors:** Anne Gregor, Beate Albrecht, Ingrid Bader, Emilia K Bijlsma, Arif B Ekici, Hartmut Engels, Karl Hackmann, Denise Horn, Juliane Hoyer, Jakub Klapecki, Jürgen Kohlhase, Isabelle Maystadt, Sandra Nagl, Eva Prott, Sigrid Tinschert, Reinhard Ullmann, Eva Wohlleber, Geoffrey Woods, André Reis, Anita Rauch, Christiane Zweier

**Affiliations:** 1 Institute of Human Genetics, Friedrich-Alexander-University Erlangen-Nuremberg, Erlangen, Germany; 2 Institut für Humangenetik, Universitätsklinikum, Universität Duisburg-Essen, Essen, Germany; 3 Department of Medical Genetics, Kinderzentrum Munich, Munich, Germany; 4 Department of Clinical Genetics, Leiden University Medical Centre, Leiden, The Netherlands; 5 Institute of Human Genetics, Rheinische Friedrich-Wilhelms-University, Bonn, Germany; 6 Institut für Klinische Genetik, Medizinische Fakultät Carl Gustav Carus, Technische Universität Dresden, Dresden, Germany; 7 Institute of Medical Genetics and Human Genetics, Charité - Universitätsmedizin Berlin, Berlin, Germany; 8 Department of Medical Genetics, Institute of Mother and Child, Warsaw, Poland; 9 Center for Human Genetics, Freiburg, Germany; 10 Centre de Genetique Humaine, Institut de Pathologie et de Genetique, Gosselies (Charleroi), Belgium; 11 Synlab Medizinisches Versorgungszentrum Humane Genetik Munich GmbH, Munich, Germany; 12 Department of Human Molecular Genetics, Max Planck Institute for Molecular Genetics, Berlin, Germany; 13 Cambridge Institute for Medical Research, Wellcome Trust/MRC Building, Addenbrooke's Hospital, Cambridge, UK; 14 Institute of Medical Genetics, University of Zurich, Zurich-Schwerzenbach, Switzerland

## Abstract

**Background:**

Heterozygous copy-number and missense variants in *CNTNAP2 *and *NRXN1 *have repeatedly been associated with a wide spectrum of neuropsychiatric disorders such as developmental language and autism spectrum disorders, epilepsy and schizophrenia. Recently, homozygous or compound heterozygous defects in either gene were reported as causative for severe intellectual disability.

**Methods:**

99 patients with severe intellectual disability and resemblance to Pitt-Hopkins syndrome and/or suspected recessive inheritance were screened for mutations in *CNTNAP2 *and *NRXN1*. Molecular karyotyping was performed in 45 patients. In 8 further patients with variable intellectual disability and heterozygous deletions in either *CNTNAP2 *or *NRXN1*, the remaining allele was sequenced.

**Results:**

By molecular karyotyping and mutational screening of *CNTNAP2 *and *NRXN1 *in a group of severely intellectually disabled patients we identified a heterozygous deletion in *NRXN1 *in one patient and heterozygous splice-site, frameshift and stop mutations in *CNTNAP2 *in four patients, respectively. Neither in these patients nor in eight further patients with heterozygous deletions within *NRXN1 *or *CNTNAP2 *we could identify a defect on the second allele. One deletion in *NRXN1 *and one deletion in *CNTNAP2 *occurred *de novo*, in another family the deletion was also identified in the mother who had learning difficulties, and in all other tested families one parent was shown to be healthy carrier of the respective deletion or mutation.

**Conclusions:**

We report on patients with heterozygous defects in *CNTNAP2 *or *NRXN1 *associated with severe intellectual disability, which has only been reported for recessive defects before. These results expand the spectrum of phenotypic severity in patients with heterozygous defects in either gene. The large variability between severely affected patients and mildly affected or asymptomatic carrier parents might suggest the presence of a second hit, not necessarily located in the same gene.

## Background

Recent data suggested that heterozygous variants or defects in *NRXN1(Neurexin 1) *or *CNTNAP2 (contactin associated protein 2)*, both genes encoding neuronal cell adhesion molecules, represent susceptibility factors for a broad spectrum of neuropsychiatric disorders such as epilepsy, schizophrenia or autism spectrum disorder (ASD) with reduced penetrance and no or rather mild intellectual impairment [1-23]. In contrast, biallelic defects in either gene were reported to result in fully penetrant, severe neurodevelopmental disorders. Strauss et al. reported on a homozygous stop mutation in *CNTNAP2 *in Old Order Amish children causing CDFE (Cortical Dysplasia - Focal Epilepsy) syndrome (MIM #610042), characterized by cortical dysplasia and early onset, intractable focal epilepsy leading to language regression, and behavioral and mental deterioration [24,25]. In a former study we reported on homozygous or compound heterozygous defects in *CNTNAP2 *or *NRXN1 *in four patients with intellectual disability and epilepsy [26], resembling Pitt-Hopkins syndrome (PTHS, MIM #610954). A possible shared synaptic mechanism that was observed in *Drosophila *might contribute to the similar clinical phenotypes resulting from both heterozygous and recessive defects in human *CNTNAP2 *or *NRXN1 *[26].

To further delineate the clinical phenotype associated with potentially recessive defects in any of the two genes, we screened a group of patients with either severe intellectual disability resembling Pitt-Hopkins syndrome or the phenotypes caused by recessive *CNTNAP2 *or *NRXN1 *defects. Additionally, we performed mutational testing in patients found to harbor heterozygous deletions in either gene.

## Methods

### Patients

Our total cohort of patients comprised four different subsets: 1. our new Pitt-Hopkins syndrome-like (PTHSL) screening group, 2. parts of our old PTHSL screening group [26], 3. a group of patients with suspected recessive inheritance, and 4. patients with known heterozygous deletions in one of the two genes. 1. The new PTHSL screening group consisted of 90 patients who were initially referred with suspected Pitt-Hopkins syndrome for diagnostic testing of the underlying gene, *TCF4*, which encodes transcription factor 4. They all had severe intellectual disability and variable additional features reminiscent of the PTHS spectrum such as dysmorphic facial gestalt or breathing anomalies. Mutational testing of *TCF4 *revealed normal results. In all of these 90 patients mutational screening of *NRXN1 *and *CNTNAP2 *was performed in the current study. Molecular Karyotyping was performed in 22 of them. This cohort does not overlap with the second subset, our old PTHSL screening group, which is a similar group of 179 patients, reported in a former study [26]. No published information on mutational screening of that group was included in the current study, but previously unpublished information on Molecular Karyotyping of 23 patients. 3. Nine patients with severe intellectual disability were referred to us specifically for *CNTNAP2/NRXN1 *testing because of suspected autosomal-recessive inheritance and/or phenotypic overlap with the previously published patients [26]. 4. In eight patients copy number changes in either *NRXN1 *or *CNTNAP2 *were identified in other genetic clinics. These were referred to us for mutational screening of the second allele. These patients had variable degrees of intellectual disability and various other anomalies. An overview on tested patients is given in Table 1. This study was approved by the ethics committee of the Medical Faculty, University of Erlangen-Nuremberg, and written consent was obtained from parents or guardians of the patients.

**Table 1:** Overview on screened patients

Patient samples used in this study	Sequencing of *NRXN1 *number of patients	Sequencing of *CNTNAP2 *number of patients	Molecular karyotyping number of patients
**1. new screening sample, n = 90**	90	90, including C1-C4	22, including N1
**2. old screening sample**[26]**,** **n=179**	published [26], results not used in this study	published [26], results not used in this study	23, not published before
3. specific testing sample*	9	9	
4. *NRXN1/CNTNAP2 *deletion group**	5, N2-N6	3, C5-C7	8, (details on arrays see Table 3)

* Patients were referred to us specifically for *NRXN1/CNTNAP2 *testing due to suspected autosomal recessive inheritance and/or phenotypic overlap with the previously published cases.

** Patients were referred to us because of copy number changes in either *NRXN1 *or *CNTNAP2 *for screening of the respective second allele.

### Molecular Karyotyping

Molecular karyotyping was performed in 45 patients without *TCF4 *mutation with an Affymetrix 6.0 SNP Array (Affymetrix, Santa Clara, CA), in accordance with the supplier's instructions. Copy-number data were analyzed with the Affymetrix Genotyping Console 3.0.2 software. In patient C3 molecular karyotyping was performed with an Affymetrix 500K array and data analysis was performed using the Affymetrix Genotyping Console 3.0.2 software.

The patients with heterozygous copy number variants (CNVs) referred for sequencing of the second allele, had been tested on different platforms. An overview on the array platforms, validation methods and segregation in the families is given in Tables 2 and 3.

**Table 2:** Molecular findings in *NRXN1*

*NRXN1*	Defect	Array Platform and details of *NRXN1/CNTNAP2 *deletion	Validation of Array data	Inheritance	Carrier parent	Other non-polymorphic CNVs	*NRXN1* sequen-cing	*CNTNAP2* sequen-cing
**N1**	*NRXN1 *deletion of exons 1-4	Affymetrix 6.0 SNP Array chr2:50.860.393-51.208.000 348 kb (230 array marker)	MLPA as reported previously [26]	paternal	healthy, normal intelligence	none	no 2^nd ^mutation	normal
**N2**	*NRXN1 *deletion of exons 1-18	Agilent 244K+customized array chr2:50.270.203-51.257.206 987 kb	customized Oligonucleotide array	maternal	learning disabilities and behavioral problems	none	no 2^nd ^mutation	normal
**N3**	*NRXN1 *deletion of exons 1-2	Agilent 244A chr2:51.011.745-51.144.527 133 kb	qPCR as reported previously [31]	maternal	healthy	21q22.3:44.534.530-44.820.473 pat dup Xp22.33:0.000.001-2.710.316 mat dup	no 2^nd ^mutation	normal
**N4**	*NRXN1 *deletion of exons 1-4	Agilent 244A chr2:50.800.974-51.286.171 425 kb	FISH analysis with BAC clones RP11-67N9 and RP11-643L22	paternal	healthy	15q26.1:88.028.337-88.072.545 mat del 16q12.1:50.773.658-51.135.179 mat dup	no 2^nd ^mutation	normal
**N5**	*NRXN1 *deletion of exons 3-4	Agilent 244A chr2:50.861.527-51.090.563, 229 kb	qPCR as reported previously [31]	paternal	muscular problems & stroke; parents consang.	none	no 2^nd ^mutation	normal
**N6**	*NRXN1 *deletion of exons 1-2	Agilent 244A chr2:51.033.865-51.496.143 462 kb	Agilent 244A of the parents	de novo		none	no 2^nd ^mutation	normal

**published biallelic defect** P3, Zweier et al. 2009 **n = 1 **[26]	*NRXN1 *deletion of exons 1-4 + p.S979X	Affymetrix 6.0 SNP Array 113 kb		parents heterozygous carriers	healthy			
**published heterozygous defects ass. with ASD** **n = 18 **[5,9,14,16,22]	15x *NRXN1 *deletion [5,14,16,22], 2x NRXN1 gain [14], 1x balanced chromosomal rearrangement disrupting *NRXN1 *[9]	12x Agilent 244K [5], 3x NimbleGen custom arrays [14], 1x Affymetrix 100 K Assay [16], 1x Affymetrix 10 K Assay [22], 66 kb-5 Mb		6x de novo [5,16,22]; 5x mat [5,14]; 4x pat [5,9]; 3x not available [5,14]		1x duplication 14q24 [14]		

mat, maternal; pat, paternal; dup, duplication; del, deletion; ass., associated; FISH, fluorescence in-situ hybridization; qPCR, quantitative Real-Time-PCR; non-polymorphic CNVs: CNVs that have not been reported in the Toronto Database of Genome Variants or have not been identified in one of our molecularly karyotyped healthy control indivuals

**Table 3:** Molecular findings in *CNTNAP2*

*CNTNAP2*	Defect	Array Platform and details of *NRXN1/CNTNAP2 *deletion	Validation of Array data	Inheritance	Carrier parent	Other non-polymorphic CNVs	*NRXN1* sequencing	*CNTNAP2* sequencing
**C1**	*CNTNAP2* c.1175_1176dup; p.D393RfsX51	Affymetrix 6.0 SNP Array, normal results for *CNTNAP2 *and *NRXN1*		paternal	healthy	chr9:9.337.920-10.207.671 mat dup chr13:19.104.340-19.477.398 mat dup	normal	no 2^nd ^mutation; MLPA normal
**C2**	*CNTNAP2 *c.2153G>A, p.W718X	Affymetrix 6.0 SNP Array, normal results for *CNTNAP2 *and *NRXN1*		not known	not known	none	normal	no 2^nd ^mutation; MLPA normal
**C3**	*CNTNAP2 *c.1083G>A, splice site (p.V361V)	Affymetrix 500 K SNP Array, normal results for *CNTNAP2 *and *NRXN1*		paternal	healthy	none	normal	no 2^nd ^mutation; MLPA normal
**C4**	*CNTNAP2 *c.1083G>A, splice site (p.V361V)	Illumina 317 K SNP Array, normal results for CNTNAP2 and *NRXN1*		maternal	healthy	**pathogenic frameshift mutation in MEF2C **(P7, Zweier et al. 2010) [28]	normal	no 2^nd ^mutation; MLPA normal
**C5**	*CNTNAP2 *deletion of exons 2-3	Affymetrix 6.0 SNP Array chr7:146.079.333-146.194.785 115 kb (69 array marker)	Affymetrix 6.0 SNP Array of the parents	maternal	healthy	none	normal, one silent variant	no 2^nd ^mutation
**C6**	*CNTNAP2 *deletion of exons 3-4	Illumina Human 660W-Quad chr7:146.144.267-146.374.539 230 kb (53 array marker)	qPCR as reported previously [32]	maternal	healthy	none	normal	no 2^nd ^mutation
**C7**	*CNTNAP2* deletion of exons 21-24	Agilent 2 × 400 K chr7:147.702.165-148.378.711 677 kb	customized Oligonucleotide array	de novo	healthy	chr7:92.394.428-92.530.356 del chr7:93.464.449-94.430.690 del, both de novo conventional karyotyping: 46,XX,der(4)t(4;10)(q25;q24), der(7)t(4;7)(q25;q32), der(10)inv(10)(p13q24)(7;10)(q32;p13), de novo	normal	no 2^nd ^mutation

**published biallelic defects** **n = 13**[24,25]	2x *CNTNAP2 *deletion of exons 2-9, homozygous [26]; 1x *CNTNAP2 *deletion of exons 5-8 + IVS10-1G>T [26]; 10x *CNTNAP2 *c.3709delG, homozygous [24,25]	2x Affymetrix 500 K/250 K Nsp SNP Array; 1x Affymetrix 6.0 SNP Array [26]; 10x no		parents heterozygous carriers				
**published heterozygous defects** **n = 12 **[1,3,7,12,21,33]	2x translocation disrupting *CNTNAP2 *[12,33], 1x inversion disrupting *CNTNAP2 *[3], 5x *CNTNAP2 *deletion [1,7,21], 4x missense variant in *CNTNAP2 *[3]	3x BAC array [7], 1x NimbleGen custom array [21], 220 kb-11 Mb		2x not reported [7], 4x inherited [3], 2x paternal [1,21], 2x de novo [3,7] 2x balanced in parent (translocation) [12,33]				

mat, maternal; pat, paternal; dup, duplication; del, deletion; ass., associated; qPCR, quantitative Real-Time-PCR; non-polymorphic CNVs: CNVs that have not been reported in the Toronto Database of Genome Variants or have not been identified in one of our molecularly karyotyped healthy control indivuals

### Mutational Screening and MLPA

DNA samples of 107 patients were derived from peripheral blood, and if sample material was limited, whole genome amplification was performed using the Illustra GenomiPhi V2 DNA Amplification Kit (GE Healthcare, Little Chalfont, Buckinghamshire, United Kingdom) according to the manufacturer's instructions. All coding exons with exon-intron boundaries of *CNTNAP2 *(NM_014141) and of isoforms alpha1, alpha2 and beta of *NRXN1 *(NM_004801; NM_001135659; NM_138735) were screened for mutations by unidirectional direct sequencing (ABI BigDye Terminator Sequencing Kit v.3; AppliedBiosystems, Foster City, CA) with the use of an automated capillary sequencer (ABI 3730; Applied Biosystems). Mutations were confirmed with an independent PCR and bidirectional sequencing from original DNA. Primer pairs and conditions were used as previously described [26]. For splice site prediction, eight different online tools were used as indicated in Table 4. Multiplex Ligation Dependent Probe Amplification (MLPA) for all coding exons of *CNTNAP2 *was performed for patients C1-C4 as described previously [26].

**Table 4:** Splice site prediction for splice donor variant c.1083G>A

Program	wild type score	mutant score
NNSplice 0.9 [34]	0.99	0.6
HSF V2.4 [35]	91.56	80.98
MaxEntScan [36]		
*Maximum Entropy Model*	8.37	3.38
*Maximum Dependence Decomposition Model*	11.88	9.78
*First-order Markov Model*	7.5	3.88
*Weight Matrix Model*	8.9	5.73
Splice Site Score Calculation [37]	8.1	5.2
Splice Site Analyzer-Tool [38]	83.27 ΔG -7.1	71.36 ΔG -4
Splice Predictor [39]	0.967	splice site not recognized
NetGene2 [40]	0.95	0.55
SplicePort [41]	1.06619	0.26169

## Results

### Molecular Testing

Mutational screening of *NRXN1 *in 90 *TCF4 *mutation negative patients and nine families with suspected recessive inheritance of severe intellectual disability did not reveal any point mutation, while in *CNTNAP2 *the heterozygous mutation c.1083G>A in the splice donor site of exon 7 was found in two patients (C3, C4). Eight prediction programs (Table 4) showed diminished splice site recognition for this mutation, which is therefore predicted to result in an in-frame loss of exon 7. This possible splice site mutation was found in one of 384 control chromosomes. Furthermore, in patient C1 the heterozygous frameshift mutation p.D393RfsX51 in exon 8 and in patient C2 the heterozygous stop mutation p.W718X in exon 14 were identified. Due to their nature and location both truncating mutations are predicted to result in mRNA decay and loss of the affected allele. For patient C2 parents were not available, but all other mutations were shown to be inherited from a healthy parent. No defect on the second allele was identified in any of these patients by sequencing and subsequent MLPA-analysis of all coding exons. In 942 controls sequenced by Bakkaloglu et al. [3], no truncating mutation in *CNTNAP2 *was found. No *CNTNAP2 *deletion was found in 667 control individuals molecularly karyotyped [26].

Molecular karyotyping with an Affymetrix 6.0 SNP Array in 45 *TCF4 *mutation negative patients revealed a heterozygous deletion within the *NRXN1 *gene in one patient (N1). The father was shown to be healthy carrier, and no mutation on the second allele was found in this patient by sequencing of all coding exons.

In three patients with *CNTNAP2 *deletions (C5-C7) and in five patients with *NRXN1 *deletions (N2-N6) we could not identify any pathogenic mutation on the second allele by sequencing all coding exons. In patient N6 and in patient C7 the deletion within *NRXN1 *or *CNTNAP2 *was shown to be *de novo*. In all other families the deletion in *CNTNAP2 *or *NRXN1 *was also identified in one of the parents.

**Figure 1:**
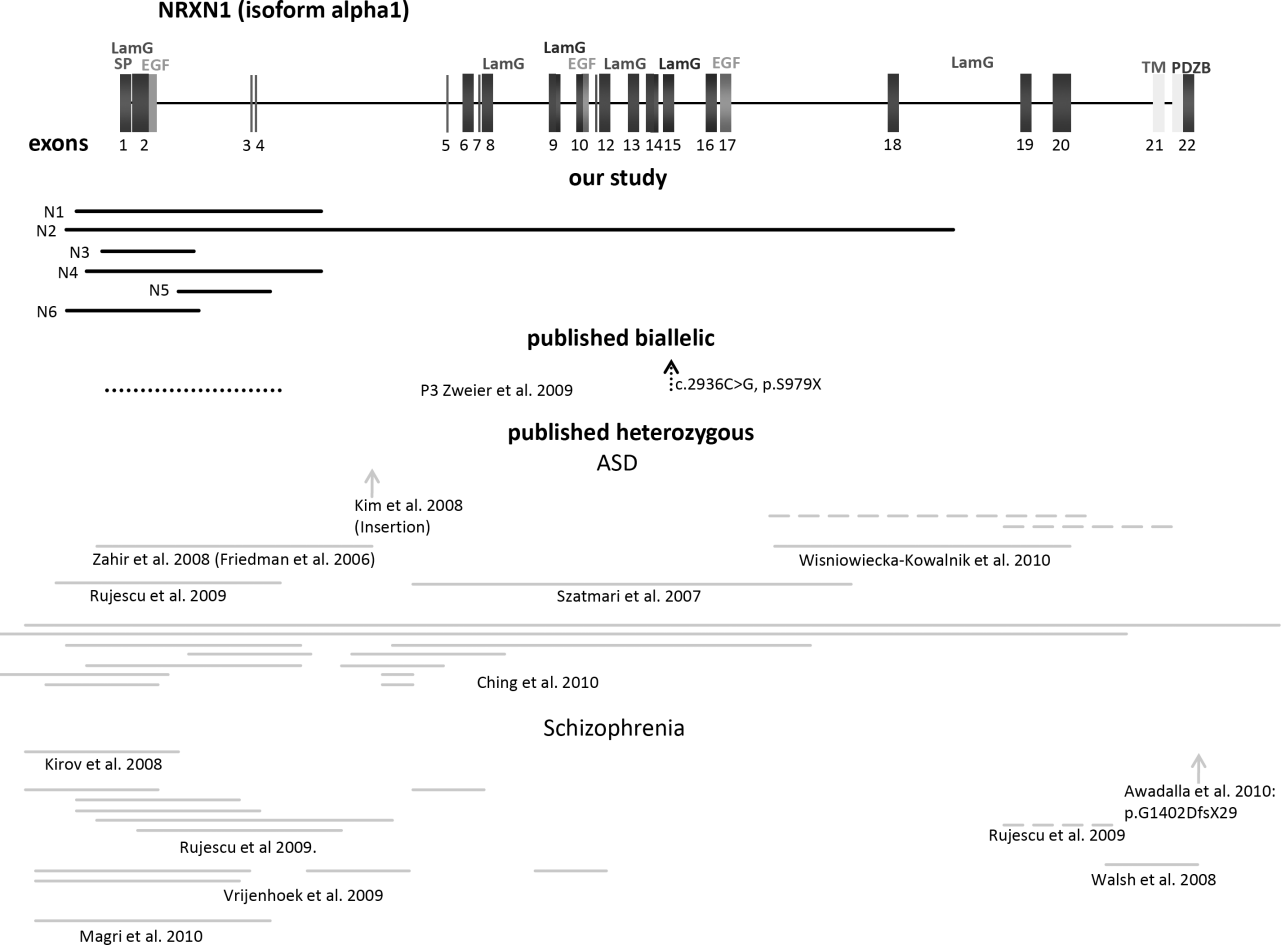
**Schematic drawing of *NRXN1 *with localization of novel and published mutations and deletions**. Schematic drawing of genomic structure of alpha 1 isoform of *NRXN1 *showing domain-coding exons and localization of mutations and deletions. Deletions found in our study are represented by black bars. Published biallelic aberrations are shown with black dotted lines, whereas heterozygous losses and gains are marked by grey solid and dashed lines, respectively. Abbreviations are as follows: SP, signal peptide; LamG, laminin-G domain; EGF, epidermal growth factor like domain; TM, transmembrane region; PDZBD, PDZ-domain binding site.

**Figure 2:**
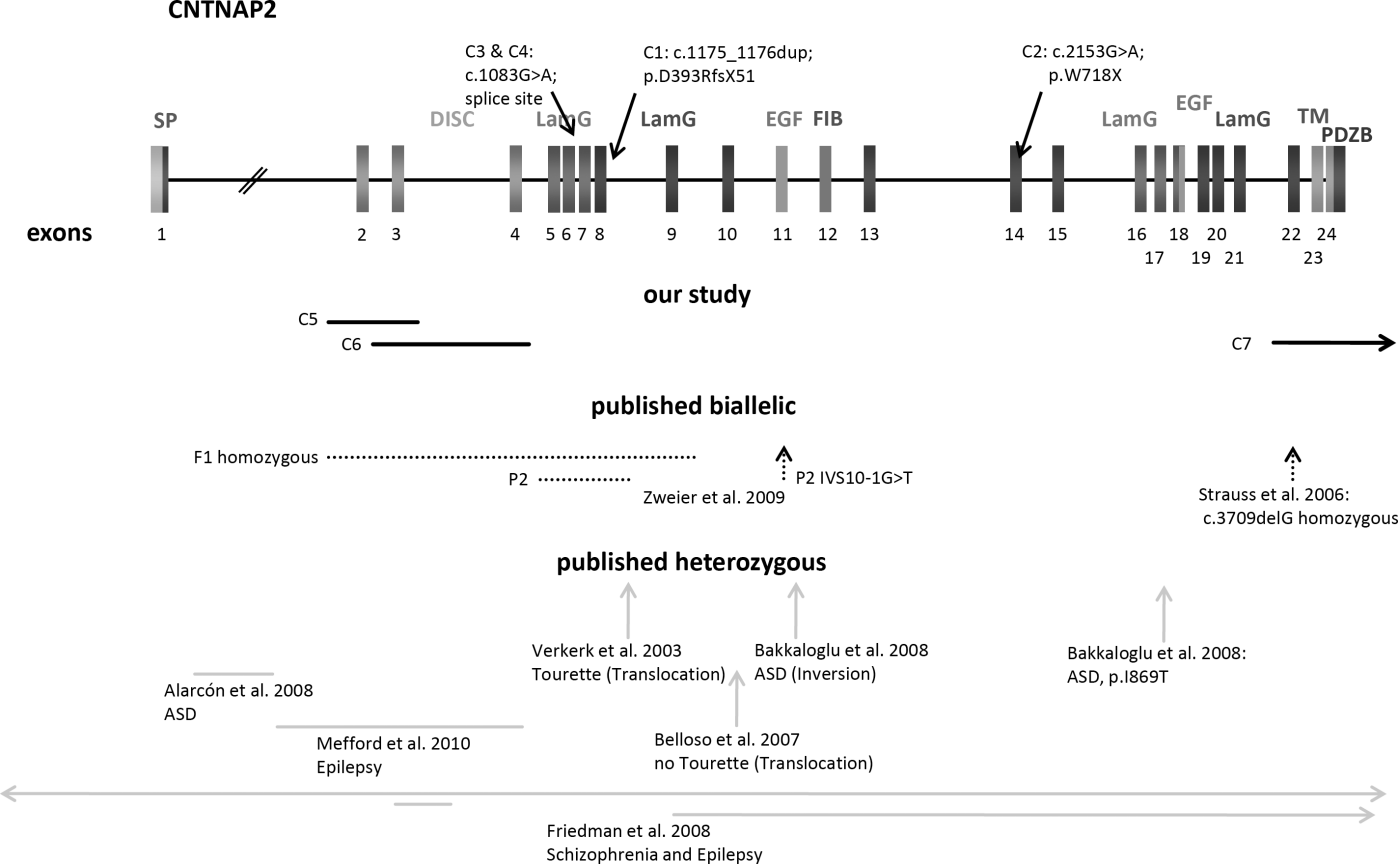
**Schematic drawing of *CNTNAP2 *with localization of novel and published mutations and deletions**. Schematic drawing of genomic structure of *CNTNAP2 *showing domain-coding exons and localization of mutations and deletions. Mutations and deletions found in our study are represented by black arrows and bars. Published biallelic aberrations are shown with black dotted lines, whereas heterozygous defects are shown in grey. Abbreviations are as follows: SP, signal peptide; DISC, discoidin-like domain; LamG, laminin-G domain; EGF, epidermal growth factor like domain; FIB, fibrinogen-like domain; TM, transmembrane region; PDZBD, PDZ-domain binding site.

In all patients with a heterozygous defect in *CNTNAP2 *we also screened *NRXN1 *and vice versa, without observing any anomalies. An overview of localization of novel and published mutations and deletions is shown in Figure 1 and 2. Mutation and array data of novel patients are shown in Tables 2 and 3.

### Clinical Findings

Four of six patients with heterozygous CNVs in *NRXN1 *were severely intellectually disabled (N1-N4). Three had epilepsy and one episodic hyperbreathing. Patients N5 and N6 were only mildly intellectually disabled and N5 additionally had various malformations like choanal atresia, anal atresia, and skeletal anomalies. All patients had absent or impaired language abilities, while motor development was normal or only mildly delayed in four of them. The deletion in patient N6 was shown to be *de novo*, in all other families one parent was shown to be carrier of the deletion. The mother of N2 was reported to have had learning difficulties, all others were reported to be healthy and of normal intelligence. However, detailed neuropsychiatric testing was not performed. Summarized clinical details of the patients are shown in Table 5.

**Table 5:** Clinical findings associated with defects in *NRXN1*

*NRXN1*	Sex & Age	ID	Speech	Age of Walking	Seizures age of onset	Birth parameters Weight, Heigth, OFC	Weight Height OFC	Behavioral anomalies/ Stereotypies	Facial dysmorphisms	Other findings
**N1**	m, 14y	Severe	at 3y: max. 10 single words, lost this function	14mo	yes	2900 g 52 cm 34 cm	P25-P50 P25-P50 P90	yes, puts objects in his mouth	large mouth, widely spaced teeth, upslanting palpebral fissures, strabism	hyperbreathing
**N2**	m, 6y	Severe	at 24mo: single words and two word combinations, receptive better than expressive	16mo	none	3740 g 51 cm 38.5 cm	Normal <P3 >P95	none	macrocephaly (also maternal and paternal), large mouth, retrogenia	muscular hypotonia, MRI: wide ventricles
**N3**	m, 3y 4mo	Severe	no active speech	14mo	none	3350 g 52 cm 35 cm	P50-P75 P75-P90 P50-P75	yes	none	none
**N4**	f, 16y	Severe	none	no	grand mal 4y	3530 g 51 cm 33 cm	P10-P25 P25-P50 <P5	yes, hand licking	broad nasal tip, pointed chin	drooling, friendly
**N5**	m, 21y	Mild	impaired	not known	grand mal, 6y (until age 11y)	3300 g 51 cm 33 cm	P3-P10 <P3 P50	none	mild facial asymmetry, small ears, broad nose, broad mouth, bushy eye brows, high arched palate, cleft lip	pectus excavatum, single transverse palmar crease, choanal atresia, anal atresia, thick finger joints, ureter stenosis, delayed bone age, spondyloptosis L5/S1
**N6**	f, 6y 3mo	Mild	2 y: first words, speech delay mainly affecting active speech	21mo	none	2820 g 50 cm 35 cm	P10-P25 P3 P10-P25	none	protruding ears	muscular hypotonia (improved), scapulae alatae, mild lordosis, tendency to diarrhea

**published biallelic defect** P3, Zweier et al. 2009 **N = 1 **[26]	f, 18y	Severe	none	2y	none	3450 g normal	P50-P75 P50-P75 P25	yes, hypermotoric behavior	broad mouth, strabism, protruding tongue	excessive drooling, developmental regression, abnormal sleep-wake-cycles, decreased deep-tendon reflexes upper extremities, hyperbreathing
**published heterozygous defects ass. with ASD** **N = 18 **[5,9,14,16,22]		7x normal [5], 3x learning problems [5,14] 2x dev. Delay [5,22], 3x mild ID [9,14,16], 2x moderate ID [5]	14x language delay [5,14,16,22]	5x motor delay [5,16]	1x yes [5]	not reported	not reported	11x ASD or Asperger syndrome [5,9,14,16,22]	11x mild dysmorphic features [5,14,16]	1x VACTERL association [5], 1x mild skeletal anomalies [16], 4x hypotonia, 2x ventricular septum defect, 3x hemangioma [5]

TOF, tetralogy of Fallot; f, female; m, male; y, year; mo, month; ASD, autism spectrum disorder; published reports on CNTNAP2 and NRXN1: only papers containing clinical data are cited; ass., associated; P, centile; ass., associated

All seven patients with heterozygous defects in *CNTNAP2 *in this study showed severe to profound intellectual disability. Speech was lacking in four patients (C1, C4-C6) and reported to be simple in C7. Patient C3 lost her speech ability at age 2.5 years. Motor impairment was also severe with no walking abilities in three patients (C4-C6), patient C7 started to walk at the age of 15 months, and patients C1 and C3 lost this function at age 2.5 - 3 years. Five patients had seizures. As far as data were available, epilepsy was of early onset and difficult to treat. At least in two of the patients episodes of hyperbreathing were reported. Congenital anomalies and malformations such as tetralogy of Fallot, pyloric stenosis, and variable other anomalies or septo-optical dysplasia were reported in patients C1 and C5, respectively. In the parents shown to be carriers, no neuropsychiatric anomalies were reported. However, detailed neuropsychiatric testing was not performed.

Summarized clinical details of the patients are shown in Table 6.

**Table 6:** Clinical findings associated with defects in *CNTNAP2*

*CNTNAP2*	Sex & Age	ID	Speech	Age of Walking	Seizures age of onset	Birth parameters Weight, Heigth, OFC	Weight Height OFC	Behavioral anomalies/ Stereotypies	Facial dysmorphisms	Other findings
**C1**	f, 8y	Severe	none	2y with aid, lost this function (3y)	yes, resist. to treatment	2430 g 45 cm not reported	<P3 <P3 <P3	hand movements	synophrys, long eyelashes, prominent columella, short philtrum, arched palate, widely spaced teeth, prominent jaw	happy, affectionate, TOF, pyloric stenosis, vesicoureteric reflux, agenesis of labia minora, hirsutism, tapering fingers
**C2**	m, 18y	Severe	?	?	complex, early onset	?	?	?		hyperbreathing, apnoe episodes
**C3**	f, 11y	Severe	few words, lost this function	2,5y, lost this function	3y	3510 g	P10 <P3 P10	yes	broad mouth, protruding tongue	develop. regression from 15 m, swallowing problems, nocturnal laughing, scoliosis, spastic tetraparesis, hyperreflexia, constipation, hyperbreathing
**C4** Zweier et al., 2010 [28]	f, 7y	Profound	none	no	3-6mo	3400 g	P5 <P2 P50	yes	broad forehead, prominent ear lobes, widely spaced teeth, tented upper lip	exotropia, heterochromasia, high pain threshold, cold feet, sleeping problems, joint hyperlaxity
**C5**	f, 2y 8mo	Profound	none	no, no crawling	none	4030 g 53 cm 38 cm	P75 P25-50		high arched palate, upslanting palpebral fissures, small teeth, prominent forehead	septo-optical dysplasia, MRI: agenesis of septum pellucidum
**C6**	f, 8y	Profound	none	no	yes, resist. to treatment	1160 g 35 cm 28 cm	<P3 <P3 <P5		mild synophrys, low set, large ears, fleshy ear lobes, thin upper lip, low frontal hairline	birth at 29^th ^week of gestation, blindness, hydrocephalus, ductus arteriosus, syndactyly toes 2-3, hypotonia, spasticity of legs, obstipation, liquid uptake by PEG tube
**C7**	f, 8y	moderate to severe	simple	15mo	none	3860 g 54 cm 34 cm	P25-P50 P50 <P5	suspected in infancy	epicanthal folds, tented upper lip, short columella, bulbous nose	overfriendliness, pubertas praecox, delayed bone age, retentive memory, excessive empathy, autoagressive behavior, flat feet

**published biallelic defects** **N = 13 **[24,25]	2x f, 1x m, 10x not reported, 1-20y	Severe	2x no, 1x single words [26], 10x yes, but regression [24,25]	2x normal, 1x not known [26], 10x 16mo-30mo [24,25]	13x yes, 4mo-30mo	not reported	<P3-normal not reported <P3-P99	8x yes [24,26], 1x tooth grinding and repetitive hand movements [26]	2x wide mouth and thick lips [26]	1x dry skin, 1x regression, 1x cerebellar hypoplasia, 3x hyperbreathing [26], 10x developmental regression with onset of seizures, 9x decreased deep tendon reflexes [24,25], 4x MRI: cortical dysplasia [24], 1x MRI: leukomalacia, 1x hepatosplenomegaly [25]
**published heterozygous** **defects** **N = 12 **[1,3,7,12,21,33]		6x not reported [1,3,21], 1x normal [7], 2x mild-moderate [3,7], 3x severe [7,12,33]	6x not reported [1,3,21], 1x normal [7], 3x speech impairment [7,12] 2x no [7,33]	11x not reported [1,3,7,12,21], 1x no [33]	5x not reported [1,3], 2x no [12,33], 5x yes [3,7,21], 0y-34y	not reported	not reported	8x yes [1,3,7]	not reported	1x multiple congenital malformations [33], 1x Gilles de la Tourette syndrome [12], 3x Schizophrenia [7]

TOF, tetralogy of Fallot; f, female; m, male; y, year; mo, month; ASD, autism spectrum disorder; published reports on CNTNAP2 and NRXN1: only papers containing clinical data are cited; ass., associated; P, centile; ass., associated

## Discussion

*NRXN1*. While the majority of the novel patients had severe intellectual disability, only two of the patients, N5 and N6, with heterozygous deletions in *NRXN1 *had mild intellectual disability as reported before for this kind of defects [5,9,11,14,16]. Additionally, patient N5 had various congenital malformations and anomalies. Interestingly, one recently published patient with a *NRXN1 *defect and no significant intellectual impairment was reported with similar malformations resembling the VACTERL spectrum [5]. Mild skeletal anomalies were also reported in the patient published by Zahir et al. [16]. A larger number of patients and therefore further delineation of the phenotype will probably clarify a possible relation of such malformations to *NRXN1 *defects. All other four patients with heterozygous *NRXN1 *deletions were severely intellectually disabled without specific further anomalies. Their phenotype resembled the patient reported with a compound heterozygous defect in this gene [26]. Except for patient N4, speech impairment was severe compared to a rather mild motor delay. Because of the severe phenotype in the patients in contrast to the normal or only mildly impaired intellectual function in the respective carrier parent, a defect of the second allele was suspected in the patients, but not found.

*CNTNAP2*. Most of the clinical aspects and the severity of intellectual disability in the herewith reported patients with heterozygous *CNTNAP2 *defects resembled those observed in patients with biallelic defects in *CNTNAP2 *reported before (Table 6). Two of the patients (C1, C3) showed language and motor regression correlating with onset of epilepsy. All others showed lacking or severely impaired speech development. However, in contrast to the published patients with recessive defects and normal or only mildly delayed motor development [24,26], all but one patients in this study also showed severe motor retardation. We could not identify a defect on the second allele in any of the novel patients. In most of the families the defect was inherited from a healthy parent. Despite a significantly higher frequency (p < 0.01, Fisher's exact test) of two truncating mutations in our cohort of 99 severely to profoundly intellectually disabled patients compared to no truncating mutation in 942 normal controls [3] definite proof that the respective mutation is fully responsible for the phenotype is so far lacking. This also applies to the other identified defects in *CNTNAP2 *or *NRXN1*.

Congenital malformations as described in patients C1 or C5 (Table 6) have not yet been reported in any other patient with a *CNTNAP2 *defect. Furthermore, the fact that the expression of the gene is restricted to the nervous system [27] does not explain these anomalies. Therefore, another genetic cause for these malformations might exist. Thus it is difficult to define if the intellectual disability is associated with the *CNTNAP2 *mutation at all in these patients. Other factors like premature complicated birth in patient C6 might contribute to impaired intellectual function. C3 and C4 carried the same splice site mutation and both showed a similar phenotype with severe intellectual disability and seizures, C3 also with breathing anomalies. In a parallel research project, a mutation in the *MEF2C *gene was identified in patient C4 and shown to be capable of causing all of her symptoms [28]. Therefore, it remains unclear if this splice mutation has a pathogenic effect at all, or only a mild effect that is masked by the severe consequences of the *MEF2C *mutation. The fact that this variant is supposed to lead to an in-frame loss of a single exon with a possibly milder effect than more deleterious defects supports the idea of no or only minor relevance of this splice mutation. Regarding the relatively high frequency of the splice site mutation in two families and one control, a founder effect might be considered, however, common regional background in these persons is not obvious.

Expanding the observations from previous studies we now found that heterozygous defects in *CNTNAP2 *or *NRXN1 *can also be seen in association with severe intellectual disability. Possible explanations might be: 1. No pathogenic relevance of the identified defect. This might indeed be the case for those patients with a "mild mutation" such as the splice-site mutation in *CNTNAP2*, or for patients with an atypical phenotype or congenital malformations. In those, the true causative defect might not be detected yet. However, published data and our data together still support a pathogenic role for both genes in neurodevelopmental disorders. 2. Inability to identify a defect on the second allele in spite of extensive screening for mutations and/or deletions. However, mutations in regulatory elements or in additional alternative isoforms cannot be excluded in any case. 3. A larger phenotypic variability associated with heterozygous defects in each gene. The finding of homozygous or compound heterozygous defects in previous patients with severe phenotypes [24-26] indicates the existence of second hits or additional major contributors. These might not necessarily be affecting the same gene. Only recently, a two-hit model for severe developmental delay in patients with a recurrent 16p12.1 microdeletion was postulated [29]. This might also be the case for microdeletions or even point mutations within a single gene as already reported for digenic inheritance in specific ciliopathies like Bardet-Biedl syndrome [30]. In four of our patients additional *de novo *or parentally inherited CNVs were identified (see Tables 2 and 3), however, the significance of these CNVs is unclear. The possible functional synaptic link between *CNTNAP2 *and *NRXN1 *[24-26] prompted us to screen *CNTNAP2 *in patients with *NRXN1 *defects and vice versa, however, without any mutation detected.

## Conclusion

We found heterozygous defects in *CNTNAP2 *and *NRXN1 *in patients with severe intellectual disability, therefore expanding the clinical spectrum associated with monoallelic defects in either gene. This large variability implicates difficulties for genetic counseling in such families. To explain the larger phenotypic variability and severity in some patients we suggest a contribution of major additional genetic factors. To identify these possible contributors and modifiers will be a great challenge for the near future.

## Competing interests

The authors declare that they have no competing interests.

## Authors' contributions

BA, IB, EKB, DH, JH, JKl, IM, EP, ST, EW, and GW acquired and provided clinical data and samples of their patients. AG, ABE, HE, KH, JKo, SN, RU, ARe, and CZ created and analysed the molecular data. ARe and ARa revised the manuscript critically for important intellectual content. CZ designed and supervised the project, and together with AG drafted the manuscript. All authors read and approved the manuscript.

## Pre-publication history

The pre-publication history for this paper can be accessed here:

http://www.biomedcentral.com/1471-2350/12/106/prepub
